# 非小细胞肺癌围手术期免疫治疗相关不良反应管理的临床诊疗建议

**DOI:** 10.3779/j.issn.1009-3419.2021.101.06

**Published:** 2021-03-20

**Authors:** 军 倪, 淼 黄, 力 张, 楠 吴, 春学 白, 良安 陈, 军 梁, 谦 刘, 洁 王, 一龙 吴, 奉春 张, 抒扬 张, 椿 陈, 军 陈, 文涛 方, 树庚 高, 坚 胡, 涛 姜, 单青 李, 鹤成 李, 永德 廖, 阳 刘, 德若 刘, 宏旭 刘, 建阳 刘, 伦旭 刘, 孟昭 王, 长利 王, 帆 杨, 跃 杨, 兰军 张, 修益 支, 文昭 钟, 宇宙 管, 潇潇 郭, 春霞 何, 少雷 李, 玥 李, 乃新 梁, 方亮 鲁, 超 吕, 玮 吕, 晓燕 斯, 锋维 谭, 汉萍 王, 江山 王, 石 阎, 华夏 杨, 惠娟 朱, 俊玲 庄, 明磊 卓

**Affiliations:** 1 100730 北京, 中国医学科学院北京协和医院 Peking Union Medical Hospital, Chinese Academy of Medical Science & Peking Union Medical College, Beijing 100730, China; 2 100142 北京, 北京大学肿瘤医院 Department of Thoracic Surgery, Peking University Cancer Hospital, Beijing 100142, China; 3 200032 上海, 复旦大学呼吸病研究所 Department of Pulmonary Medicine, Zhongshan Hospital, Fudan University, Shanghai 200032, China; 4 100853 北京, 中国人民解放军总医院 The First Medical Center of Chinese PLA General Hospital, Beijing 100853, China; 5 102206 北京, 北京大学国际医院 Peking University International Hospital, Beijing 102206, China; 6 300052 天津, 天津医科大学总医院, 天津市肺癌研究所 Tianjin Lung Cancer Institute, Tianjin Medical University General Hospital, Tianjin 300052, China; 7 100021 北京, 中国医学科学院肿瘤医院 State Key Laboratory of Molecular Oncology, National Cancer Center, National Clinical Research Center for Cancer, Cancer Hospital, Chinese Academy of Medical Sciences and Peking Union Medical College, Beijing 100021, China; 8 510000 广州, 广东省人民医院 Department of Pulmonary Cancer, Guangdong Lung Cancer Institute, Guangdong Provincial People's Hospital, Guangdong Academy of Medical Sciences, Guangzhou 510000, China; 9 350001 福州, 福建医科大学附属协和医院 Department of Thoracic Surgery, Fujian Medical University Union Hospital, Fuzhou 350001, China; 10 300052 天津, 天津医科大学总医院 Department of Lung Cancer Surgery, Tianjin Medical University General Hospital, Tianjin Key Laboratory of Lung Cancer Metastasis and Tumor Microenvironment, Tianjin Lung Cancer Institute, Tianjin 300052, China; 11 200030 上海, 上海市胸科医院 Department of Thoracic Surgery, Shanghai Chest Hospital, Shanghai Jiao Tong University, Shanghai 200030, China; 12 310003 杭州, 浙江大学医学院附属第一医院 Department of Thoracic Surgery, The First Affiliated Hospital, Zhejiang University School of Medicine, Hangzhou 310003, China; 13 710038 西安, 空军军医大学第二附属医院（唐都医院） Department of Thoracic Surgery, Tangdu Hospital, Fourth Military Medical University, Xi'an 710038, China; 14 200025 上海, 上海交通大学医学院附属瑞金医院 Department of Thoracic Surgery, Ruijin Hospital, Shanghai Jiao Tong University School of Medicine, Shanghai 200025, China; 15 430022 武汉, 华中科技大学同济医学院附属协和医院 Department of Thoracic Surgery, Union Hospital, Tongji Medical College, Huazhong University of Science and Technology, Hubei 430022, China; 16 100029 北京, 中日友好医院 China-Japan Friendship Hospital, Beijing 100029, China; 17 110001 沈阳, 中国医科大学肿瘤医院 辽宁省肿瘤医院 Department of Thoracic Surgery, Cancer Hospital of China Medical University, Liaoning Cancer Hospital & Institute, Shenyang 110001, China; 18 130012 长春, 吉林省肿瘤医院 Department of Thoracic Surgery-1, Jilin Cancer Hospital, Changchun 130012, China; 19 610041 成都, 四川大学华西医院 19 Department of Cardiovascular and Thoracic Surgery, West China Hospital, Sichuan University, Chengdu 610041, China; 20 300060 天津, 天津医科大学附属肿瘤医院 Department of Lung Cancer, Tianjin Medical University Cancer Institute and Hospital, National Clinical Research Center for Cancer, Key Laboratory of Cancer Prevention and Therapy, Tianjin's Clinical Research Center for Cancer, Tianjin 300060, China; 21 100044 北京, 北京大学人民医院 Peking University People's Hospital, Beijing 100044, China; 22 510060 广州, 中山大学附属肿瘤医院 Department of Thoracic Surgery, Sun Yat-Sen University Cancer Center, Guangzhou 510060, China; 23 100053 北京, 首都医科大学宣武医院 Department of Thoracic Surgery, Xuanwu Hospital, Capital Medical University, Beijing 100053, China

**Keywords:** 肺肿瘤, 围手术期免疫治疗, 免疫相关不良事件, 诊疗建议, Lung neoplasms, Perioperative immunotherapy, Immune checkpoint inhibitor related adverse events, Clinical recommendation

## Abstract

**背景与目的:**

肺癌围手术期治疗（术前新辅助治疗及术后辅助治疗）作为手术的重要辅助手段，已成为非小细胞肺癌（non-small cell lung cancer, NSCLC）全程管理中越来越重要的环节。近年来，小规模临床研究发现免疫新辅助治疗主要病理缓解率明显提升，甚至达到完全病理缓解，有望成为NSCLC治疗的重要组成部分。然而，免疫新辅助治疗带来疗效和生存获益，同时，其相关严重不良反应（延误手术、丧失手术机会、死亡等）备受关注。本诊疗建议目的是针对免疫检查点抑制剂相关不良反应（immune-related adverse event, irAE）形成适合国内医疗现状的诊疗方案。

**方法:**

本文由胸外科专家、肿瘤学专家、胸内科专家以及irAE相关科室专家（消化内科、呼吸内科、心血管内科、感染内科、血液内科、内分泌科、风湿免疫科、神经内科、皮肤科和急诊科）共同完成本诊疗意见的制定工作。专家充分参考irAE指南、胸外科公开发表的大型临床研究数据以及国内医生的临床实战经验和公开发表个案，多学科反复讨论，形成针对围手术期免疫治疗相关不良反应的诊疗建议。

**结果:**

本诊疗意见涵盖irAE相关的预防、评估、检查、治疗和监测全过程，以便全面、有效的指导临床工作。

**结论:**

围手术期irAE管理是肺癌免疫围手术期治疗的重要组成部分，随着免疫围手术期治疗的不断发展，未来需要更多的研究，以优化围手术irAE的诊疗。

## Abstract (en)

**Background andobjective:**

Perioperative treatment has become an increasingly important aspect of the management of patients with non-small cell lung cancer (NSCLC). Small-scale clinical studies performed in recent years have shown improvements in the major pathological remission rate after neoadjuvant therapy, suggesting that it will soon become an important part of NSCLC treatment. Nevertheless, neoadjuvant immunotherapy may be accompanied by serious adverse reactions that lead to delay or cancelation of surgery, additional illness, and even death, and have therefore attracted much attention. The purpose of the clinical recommendations is to form a diagnosis and treatment plan suitable for the current domestic medical situation for the immune-related adverse event (irAE).

**Methods:**

This recommendation is composed of experts in thoracic surgery, oncologists, thoracic medicine and irAE related departments (gastroenterology, respirology, cardiology, infectious medicine, hematology, endocrinology, rheumatology, neurology, dermatology, emergency section) to jointly complete the formulation. Experts make full reference to the irAE guidelines, large-scale clinical research data published by thoracic surgery, and the clinical experience of domestic doctors and publicly published cases, and repeated discussions in multiple disciplines to form this recommendation for perioperative irAE.

**Results:**

This clinical recommendation covers the whole process of prevention, evaluation, examination, treatment and monitoring related to irAE, so as to guide the clinical work comprehensively and effectively.

**Conclusion:**

Perioperative irAE management is an important part of immune perioperative treatment of lung cancer. With the continuous development of immune perioperative treatment, more research is needed in the future to optimize the diagnosis and treatment of perioperative irAE.

2015年以来以程序性死亡因子-1（programmed cell death-1, PD-1）单抗，程序性死亡因子-1配体（programmed cell death ligand-1, PD-L1）单抗为代表的免疫检查点抑制剂（immune checkpoint inhibitors, ICIs）在晚期非小细胞肺癌（non-small cell lung cancer, NSCLC）治疗中的数据不断公布^[1-3]^，陆续在多个国家/地区获批二线、后线及一线治疗的适应证，开启了NSCLC免疫治疗时代。2017年PACIFIC研究^[4]^的结果表明，将免疫治疗进一步前移，应用于不可切除Ⅲ期NSCLC患者化放同步治疗后的维持治疗，取得了较传统放化疗更优的生存，成为Ⅲ期不可切除NSCLC的标准治疗模式，改变了临床实践。为了提高局部进展期患者治愈率和延长患者的无病生存期（disease free survival, DFS），围手术期免疫治疗的临床研究正在陆续开展^[5]^。可切除NSCLC中新辅助免疫治疗杀伤肿瘤同时，能够触发机体释放更多肿瘤抗原激活T细胞，同时活化T细胞通过血管和淋巴管达到微病灶，引发更大范围的抗肿瘤免疫反应，从而达到降期、提高R0切除率、控制微卫星灶、提高总生存期（overall survival, OS）率等新辅助治疗目标。新辅助的治疗模式包括免疫单药、双免疫治疗、化疗联合/序贯免疫治疗、放化疗联合免疫治疗，结果陆续发表；术后辅助治疗的Ⅱ期/Ⅲ期临床研究也正在进行中，结果尚未知晓。其中，NADIM、SAKK 16/14等围手术期化疗联合免疫治疗的Ⅱ期研究^[6, 7]^表明，化疗联合免疫治疗在早期/局部晚期NSCLC患者新辅助治疗期间达到的效果主要表现在，主要病理缓解（major pathologic response, MPR）率可达60%-85%，病理完全缓解（pathologic complete response, pCR）率可达18.2%-71.4%。并且，NSCLC新辅助治疗的pCR和MPR与OS有显著的相关性^[8]^，可以预见免疫围手术期治疗模式未来将有良好的应用前景。

然而，在临床获益的同时，却也避免不了免疫治疗相关毒副反应的增加以及少数情况下可能危及生命，造成不能手术、延迟手术及增加术后并发症的可能^[9]^。肺癌辅助顺铂评估协作组（Lung Adjuvant Cisplatin Evaluation, LACE）和NSCLC Collaborative Group先后进行的荟萃分析^[9, 10]^显示，新辅助治疗降低13%死亡风险，辅助化疗将患者的5年生存率提升了5.3%（HR=0.89），但3级-4级的不良反应高达66%。虽然免疫联合化疗与单纯化疗相比，绝大多数3级-4级不良反应发生率没有显著意义的上升^[11]^，但免疫相关不良反应（immune-related adverse events, irAE），尤其是免疫相关肺炎、心脏毒性、消化道毒性及其他少见但严重毒性等已经严重影响患者的预后^[12]^。由此，临床上迫切需要更精细化的免疫相关不良反应的管理，其具体的步骤应体现在基线检查、诊断与鉴别诊断以及多学科联动的管理模式。肺癌治疗已经进入免疫治疗时代，胸部外科在肺癌免疫治疗临床应用以及不良反应管理方面处于至关重要的地位；特别是在综合性医疗单位的多科联动式管理模式中，胸部外科所起到的作用将不容忽视。随着业界对于免疫治疗相关不良反应的认识，尤其是围手术期免疫相关不良反应的关注与认识不断增加，对于相应的不良反应管理也提出更高要求。目前，在已有的研究数据和循证医学证据尚且不能满足临床需要的情况下，肺癌诊疗领域的医生，特别是胸部外科医生正面临着知识结构重组、专业延展性增加，纵深度加强等诸多挑战。面对这些痛点和挑战，我们综合了多学科意见，针对性地推出了这篇诊疗建议，旨在帮助大家梳理相关知识结构的同时，引发更多思考，不断完善肺癌围手术期免疫相关不良反应的诊疗理念，为临床工作提供更多有效的指导。

## 1 概述

目前对于免疫相关不良反应的具体发生机制尚不十分明确，一些主要的潜在机制包括T细胞对抗存在于肿瘤和正常组织上的抗原的活性增强；已存在的自身免疫抗体；炎症细胞因子水平增高；细胞毒性T淋巴细胞相关抗原4（cytotoxic T lymphocyte associated antigen-4, CTLA-4）抗体与表达CTLA-4抗体的正常组织直接结合的成分介导的免疫反应增强^[13]^。然而，激活的免疫系统也可能会攻击人体正常的器官系统，引起一系列的irAE，常见的irAE包括皮肤毒性、内分泌毒性、肝脏毒性、胃肠道毒性、肺毒性、骨骼肌肉毒性、输液反应，少见的irAE包括神经毒性、血液毒性、肾脏毒性、心脏毒性、眼毒性等。

基于免疫检查点抑制剂的独特作用机制，irAE的发生率、严重程度及不良反应类型也有别于传统的化疗。据文献^[14]^报道，在接受免疫治疗患者中，任何级别和≥3级不良事件（adverse event, AE）的发生率显著低于单纯化疗（任何级别AE：65.8% *vs* 85.2%，OR=0.35；≥3级AE：16.5% *vs* 41.1%，OR=0.26），且因AE中断治疗的比例（6.4% *vs* 10.8%, OR=0.55, 95%CI: 0.39-0.78）以及因治疗相关AE死亡的比例（0.87% *vs* 1.28%, OR=0.67, 95%CI: 0.46-0.98）也均较低。另外，在免疫治疗中，最常见的irAE是腹泻、甲状腺功能减退、天门冬氨酸氨基转移酶升高、白癜风、丙氨酸氨基转移酶升高；最常见的3级以上irAE是天门冬氨酸转氨酶升高、丙氨酸氨基转移酶升高、肺炎、腹泻、结肠炎^[15]^。irAE通常出现在治疗后的数周至数月之间，持续时间较长，且可贯穿整个治疗过程，甚至在治疗结束后出现。

围手术期免疫治疗目前有多项Ⅲ期临床研究正在积极探索，包括CheckMate 816、KEYNOTE-671、IMpower030、AEGEAN等。在大部分既往Ⅰ期/Ⅱ期研究的主要研究终点中，新辅助免疫治疗的MPR为19%-45%，双免疫联合MPR为33%，免疫联合化疗MPR高达85%。虽然从前期的Ⅰ期/Ⅱ期临床研究中看到了围手术期免疫治疗的获益潜力，但同时也观察到了一些问题，从各项研究公布的数据^[6, 16-18]^中可以看到有一部分患者因不良反应或其他原因导致手术时机延误，手术方式转换，手术效果降低，住院时间延长，患者的经济负担增加，严重者可导致围手术期并发症发生率和病死率的上升。因此，对于接受围手术期免疫治疗的肺癌患者，irAE的精细化管理需要早期识别和应用免疫抑制和/或免疫调节剂及时干预。因此，中国临床肿瘤学会（Chinese Society of Clinical Oncology, CSCO）、美国国立综合癌症网络（National Comprehensive Cancer Network, NCCN）等权威指南均指出，预防、评估、检查、治疗和监测五个环节在irAE管理的整体过程中必不可少。

### 1.1 irAE预防

#### 1.1.1 特殊人群的免疫治疗

免疫治疗在可手术期NSCLC的应用目前处于探索之中，尚需更高级别循证医学证据支持。虽然已完成的研究结果令人振奋，但这些研究纳入病例数量有限，表现出的高疾病控制率需要未来大型临床研究的结果进一步证实。因此，在临床实践过程中，需充分评估患者的可能获益与潜在风险，尤其是在合并自身免疫性疾病、有器官功能障碍、器官抑制等特殊人群中，应用免疫治疗应当更加谨慎，需充分评估围手术期免疫治疗的安全性和手术本身的安全性。①存在自身免疫性疾病患者：ICIs治疗可导致基础疾病复发或严重活动^[19, 20]^，也可诱发新发irAE出现^[21]^。因此，建议对于这部分人群的免疫抑制情况由专科医生进行密切监测；在启动免疫治疗之前，尽量把泼尼松的剂量控制在 < 10 mg/d或其等效剂量。自身免疫性神经系统疾病患者或危及生命的自身免疫性疾病的患者，尤其是免疫抑制药物不能控制或需要大剂量免疫抑制药物控制病情的患者，不适合免疫治疗；②接受过器官移植或造血干细胞移植患者：接受免疫治疗会导致移植物抗宿主病（graft-versus-host disease, GVHD）或移植器官衰竭，其中肾移植患者GVHD发病率约为50%，肝移植患者为44%，心脏移植患者为25%^[22]^。因此在启动免疫治疗前，需要和患者及移植外科医师充分讨论；既往接受过实体器官移植，且发生移植物排斥时有可行替代治疗方案的患者，可能是免疫治疗的适应证（无移植排斥的证据，且处于免疫抑制的维持治疗阶段）；③慢性病毒感染：因ICIs与慢性病毒感染间相互作用尚不清楚，因此临床试验中排除了乙型肝炎病毒（hepatitis B virus, HBV）、丙型肝炎病毒（hepatitis C virus, HCV）、人类免疫缺陷病毒（human immunodeficiency virus, HIV）慢性病毒感染患者。目前认为，ICIs治疗HBV、HCV感染的肝细胞肝癌患者，安全性和疗效与无感染患者相当^[23]^。对于HIV感染患者非ICIs治疗禁忌症，不良事件发生率与疗效相当^[24]^，少数个案报道^[25]^认为HIV感染患者CD4^+^ T淋巴细胞明显下降，免疫系统缺陷，ICIs治疗难以起效并可能诱发免疫重建炎症综合征。

#### 1.1.2 围手术期患者的选择

免疫检查点抑制剂（PD-1/L1或CTLA-4单抗等）已经证实可用于治疗局部晚期或转移性NSCLC，针对可手术期NSCLC的辅助/新辅助免疫治疗的临床试验正在开展当中（表 1，表 2）。

**1:** 新辅助免疫联合或不联合化疗治疗可切除NSCLC的Ⅰ期/Ⅱ期临床试验汇总 Summary of phase Ⅰ/Ⅱ clinical trials of neoadjuvant immunization combined with or without chemotherapy for resectable NSCLC

研究名称	研究人群	治疗方案	例数	组织学类型	EGFR	ALK	PD-L1	主要结果
CheckMate 159^[26]^	Ⅰ-Ⅲa	Nivolumab×2→S	22	鳞癌， 非鳞癌	/	/	/	MPR：45% pCR：15%
LCMC3^[16]^	Ⅰb-Ⅲa	Atezolizumab×2→S→Atezolizumab×1 y	101	鳞癌， 非鳞癌	WT	WT	+/-	MPR：19% pCR：5%
NADIM^[27]^	Ⅲa	Nivolumab+CT×3→S→Nivolumab×1 y	46	鳞癌， 非鳞癌	WT	WT	+/-	MPR：80% pCR：75%
NEOSTAR^[28]^	Ⅰ-Ⅲa	Nivolumab *vs* Nivolumab+Ipilimumab×3→S	44	鳞癌， 非鳞癌	/	/	/	MPR: 17% *vs* 33%（ITT） pCR: 9% *vs* 29%（ITT）
NCT03366766^[29]^	Ⅰ-Ⅲa	Nivolumab+CT×3→S	13	鳞癌， 非鳞癌	WT	WT	+/-	MPR：85% pCR：38%
SAKK 16/14^[7]^	Ⅲa	CT×3→Durvalumab×2→S→ Durvalumab×1 y	68	鳞癌， 非鳞癌	WT	WT	+/-	MPR：60% pCR：18.2%
ChiCTR-OIC-17013726^[18]^	Ⅰb-Ⅲb	Sintilimab×2→S→Sintilimab±CT/CT±RT	40	鳞癌， 非鳞癌	WT	WT	+/-	MPR：40.5% pCR：16.2%
NCT02716038^[30]^	Ⅰb-Ⅲa	Atezolizumab+CT×4→S	39	鳞癌， 非鳞癌	/	/	+/-	MPR：57% pCR：33%
CT：化疗；MPR：主要病理缓解；pCR：病理完全缓解；S：手术；ITT：意向治疗人群；WT：野生型；EGFR：表皮生长因子受体；ALK：间变性淋巴瘤激酶；PD-L1：程序性死亡因子-1；NSCLC：非小细胞肺癌								

**2:** 正在进行的可切除NSCLC新辅助免疫联合化疗的Ⅲ期临床试验汇总 Summary of phase Ⅲ clinical trials of neoadjuvant immunotherapy combined with chemotherapy in resectable NSCLC

研究名称	研究人群	治疗方案	例数	组织学类型	EGFR	ALK	PD-L1	预计完成时间
IMpower030 （NCT03456063）	Ⅱ-Ⅲb	CT+Atezolizumab/Placebo×4→S→ Atezolizumab/Placebo×1 y	374	鳞癌， 非鳞癌	WT	WT	+/-	2025年3月
AEGEAN （NCT03800134）	Ⅱa-Ⅲb	CT+Durvalumab/Placebo×3→S→Durvalumab/Placebo×1 y	300	鳞癌， 非鳞癌	WT/m	WT/m	+/-	2024年1月
KEYNOTE-671 （NCT03425643）	Ⅱ-Ⅲb	CT+Pembrolizumab/Placebo×4→S→ Pembrolizumab/Placebo×1 y	786	鳞癌， 非鳞癌	/	/	+/-	2026年6月
CheckMate 77T （NCT04025879）	Ⅱa-Ⅲb	CT+Nivolumab/Placebo→S→ Nivolumab/Placebo×1 y	452	鳞癌， 非鳞癌	WT	WT	+/-	2024年9月
CheckMate 816 （NCT02998528）	Ⅰb-Ⅲa	CT+Nivolumab×3→S *vs* CT×3→S	350	鳞癌， 非鳞癌	/	/	+/-	2023年5月
m：突变								

新辅助免疫治疗临床试验主要集中在早期（Ⅰb期-Ⅲb期）NSCLC患者。由于目前ICIs在新辅助阶段的应用仍未写入指南，根据各地医政管理的要求，需要在病例中完善必要的文书告知工作，并在药学部和医务处进行超适应症备案。注册临床研究入组的患者可以按照研究的入组筛选流程进行。目前的临床实践倾向于选择高T分期或者多发N2转移及融合状N2转移为新辅助免疫治疗对象，更早期的病例依然在探索中。

NCCN指南中对于完全切除（R0）的NSCLC，推荐Ⅱa期-Ⅲa期的患者进行术后辅助治疗（化疗、放疗或靶向治疗），Ⅰa期NSCLC明确不建议辅助化疗，而Ⅰb期合并有高危因素的肺癌，可考虑行术后辅助化疗，但缺乏高级别证据的支持。目前正在进行的术后辅助免疫治疗的临床试验，也正是基于指南的推荐，主要是纳入完全切除的Ⅰb期-Ⅲa期NSCLC（表 3）。

**3:** 正在进行的可切除NSCLC术后辅助免疫治疗的Ⅲ期临床试验汇总 Summary of phase Ⅲ clinical trials of adjuvant immunotherapy after resectable NSCLC

研究名称	研究人群	治疗方案	例数	组织学类型	EGFR	ALK	PD-L1	预计完成时间
IMpower010 （NCT02486718）	Ⅰb-Ⅲa	CT×4 →Atezolizumab/Placebo×1 y	1, 280	鳞癌， 非鳞癌	/	/	+/-	2027年12月
ALCHEMIST-nivo/ANVIL （NCT02595944）	Ⅰb-Ⅲa	Nivolumab/Observation×1 y±CT/RT	903	鳞癌， 非鳞癌	WT	WT	+/-	2024年7月
PEARLS/KEYNOTE-091 （NCT02504372）	Ⅰb/Ⅱ-Ⅲa	Pembrolizumab/Placebo×1 y±CT	1, 177	鳞癌， 非鳞癌	/	/	+/-	2024年2月
ADJUVANT BR.31 （NCT02273375）	Ⅰb-Ⅲa	Durvalumab/Placebo×1 y	1, 360	鳞癌， 非鳞癌	/	/	+/-	2024年1月
ALCHEMIST Chemo-IO （NCT04267848）	Ⅰb-Ⅲa	CT×4 *vs* CT×4+Pembrolizumab×1 y *vs* CT×4→Pembrolizumab×1 y	1, 263	鳞癌， 非鳞癌	WT	WT	+/-	2024年12月
RT：放疗								

对于不完全切除（R1或R2）的Ⅰb期之后的NSCLC，若不考虑二次手术，也可以考虑在术后辅助治疗之后，进行免疫维持治疗。由于ICIs在辅助阶段的应用仍未写入指南，目前仍不是常规治疗，根据各地医政管理的要求，需要在病例中完善必要的文书告知工作，并在药学部和医务处进行超适应证备案。注册临床研究入组的患者可以按照研究的入组筛选流程进行。

### 1.2 irAE评估与检查

免疫治疗前的评估与常规筛查是irAE管理中最重要的一部分，有助于我们筛选出特殊人群，早期识别和干预。在开始ICIs治疗之前，医师必须评估患者发生irAE的易感性，主要包括：现病史、既往史（特别是自身免疫性疾病病史、免疫缺陷疾病病史、特殊感染病史）、个人史、家族史、一般状况、基线实验室检验和影像学检查[胸腹盆计算机断层扫描（computed tomography, CT）、头颅磁共振成像（magnetic resonance imaging, MRI）等]（表 4）。所有患者都应该在治疗开始前被告知免疫治疗潜在的不良反应。在出现不良反应时，患者应该直接向治疗团队报告症状。一旦出现irAE，需要及时治疗，防止加重或恶化。

**4:** 基线评估 Baseline assessment

检查项目	Ⅰ级推荐	Ⅱ级推荐	Ⅲ级推荐
临床评估^a^	· 体格检查 · 自身免疫疾病或器官特异性疾病、内分泌疾病或感染性疾病 · 神经系统评估 · 排便习惯 · 吸烟史、家族史、妊娠状况 · 基线用药情况		
影像学评估^b^	· 胸部、腹部（盆腔）增强CT	· 全身PET/CT	· 根据临床指征进行头颅MRI、全身骨扫描
一般血液学检验^c^	· 血常规 · 血生化 · 凝血 · 心肌酶 · 尿常规 · 便常规 · 炎症指标	· 血糖升高者需完善尿酮体、糖化血红蛋白、胰岛素、C肽	· 根据全身情况完善IAA、ICA、GAD-Ab
病毒学检验	· 乙肝五项 · HIV-Ab、TP-Ab · CMV-DNA · EBV-DNA · 新型冠状病毒抗体（IgM+IgG）		
自身抗体^d^	· 抗核抗体谱 · 抗中性粒细胞胞浆抗体谱 · 类风湿相关抗体 · 抗乙酰胆碱酯酶抗体 · 抗Hu/Yo/Ri抗体（血+脑脊液）（针对小细胞肺癌患者）		
皮肤	· 若出现新发皮肤病变或原有皮肤病变加重，需检查皮肤和黏膜（结膜、口腔黏膜、鼻黏膜、肛周黏膜等）		
胰腺	· 血淀粉酶、脂肪酶	· 若有症状，考虑腹部增强CT（胰腺薄扫）或MRCP	
甲状腺^e^	· 甲状腺功能检测：TSH、fT4、fT3	· 若基线甲状腺功能异常，检查TT3、TT4、fT3、fT4、TPO、TgAb、TRAb	
肾上腺/垂体^e^	· F（晨起首选，8 am），ACTH（8 am）	· 若基线检查异常，完善性激素六项（LH、FSH、T、P、E2、PRL、IGF-1；垂体MRI	
肺	· 血氧饱和度（静息和活动） · 术前常规行肺功能检查，高危患者行动脉血气分析检查	· 推荐行6分钟步行试验	
心血管	· 心电图检查 · 心脏彩超	· 对于基线异常或有症状患者，定期监测，根据需要与心内科会诊进行个体化随访	
骨骼肌肉		· 根据需要进行关节检查/功能评估	· 根据病情，考虑CRP、ESR、CK、肌酶谱、抗核抗体谱、类风湿因子、抗环瓜氨酸多肽抗体检查 · 风湿免疫科会诊
神经系统		· 根据需要进行神经系统检查/功能评估	· 根据需要与神经内科会诊进行个体化评估、随访
^a^：需要详细了解患者的症状、诊疗经过、治疗相关不良反应，既往史（特别是自身免疫性疾病、免疫缺陷性疾病、结核病、病毒性肝炎、器官移植等）、过敏史、家族史（特别是自身免疫性疾病或免疫缺陷性相关家族史）；询问是否具有免疫系统疾病相关的症状和体征，包括脱发、光过敏、蝶形红斑、盘状红斑、猖獗龋齿、反复发作的口腔溃疡和（或）外阴溃疡、葡萄膜炎、眼干、口干、关节痛、关节肿胀、炎性腰背痛、肌痛、肌无力、黏液脓血便等；^b^：影像学检查：胸腹部增强CT、头颅增强MRI、全身骨显像，评估原发灶情况及有无远处转移；推荐行全身PET/CT进行分期；^c^：血常规、血生化（AST、ALT、ALP、GGT、TBil、DBil、TP、Alb、前白蛋白、LDH、Cr、BUN、Glu、K、Na、Cl、Ca、P、CO_2_结合力、UA、AMY、LIP）、凝血（PT、APTT、FIb、INR、D-dimer）、心肌酶（肌红蛋白、CK、CK-MB、cTnI、BNP/NT-proBNP）、尿常规（尿蛋白、红细胞）、便常规（潜血、红细胞、白细胞）；炎症指标：CRP、ESR、白介素-6、白介素-8、白介素-10、肿瘤坏死因子-*α*、铁蛋白；^d^：自身抗体：ANA、抗ds-DNA、抗RNP、抗SSA、抗SSB、抗Scl-70、抗Jo-1、AMA-M2；抗中性粒细胞胞浆抗体；抗环瓜氨酸多肽抗体、抗核周因子、抗角蛋白抗体、类风湿因子；^e^：内分泌相关指标：TSH、FT3、FT4、A-Tg、A-TPO；ACTH（8 am）、F（8 am）；FSH、LH、T、E2、P、PRL；IGF-1、GH。 PET/CT：正电子发射计算机断层显像；MRI：核磁共振；CT：电子计算机断层扫描；IAA：胰岛素自身抗体；ICA：胰岛细胞抗体；GAD-Ab：谷氨酸脱羧酶抗体；HIV-Ab：人类缺陷病毒抗体；TP-Ab：梅毒螺旋体抗体；CMV-DNA：巨细胞病毒核酸；EBV-DNA：EB病毒核酸；TSH：促甲状腺激素；fT4：游离甲状腺素；fT3：游离三碘甲状原氨酸；TT4：总甲状腺素；TT3：总三碘甲状原氨酸；TPO：抗过氧化物酶抗体；TgAb：抗甲状腺球蛋白抗体；TRAb：促甲状腺素受体抗体；F：血皮质醇；ACTH：促肾上腺皮质激素；LH：黄体生成素；FSH：卵泡刺激素；T：睾酮；P：孕酮；E2：雌二醇；PRL：催乳素；IGF-1：胰岛素样生长因子；CRP：C反应蛋白；ESR：血沉；CPK：肌酸磷酸激酶；AST：谷草转氨酶；ALT：谷丙转氨酶；ALP：血清碱性磷酸酶；GGT：γ-谷氨酰转肽酶；TBil：总胆红素；DBil：直接胆红素；TP：总蛋白；Alb：白蛋白；LDH：乳酸脱氢酶；Cr：肌酐；BUN：尿素氮；Glu：血糖；K：血钾；Na：血钠；Cl：血氯；Ca：血钙；P：血磷；UA：尿酸；AMY：淀粉酶；LIP：脂肪酶；PT：凝血酶原时间；APTT：活化部分凝血活酶时间；FIb：纤维蛋白原；D-dimer：D-二聚体；CK：肌酸激酶；CK-MB：肌酸激酶同工酶；cTnI：心肌肌钙蛋白I；BNP：B型钠尿肽；NT-proBNP：氨基末端脑钠肽前体；AMA-M2：抗线粒体抗体M2亚型			

#### 1.2.1 新辅助治疗之后的术前评估

① 询问病史及术前体格检查，对伴有高血压、糖尿病、冠心病患者，进行相关治疗，待病情稳定后可考虑手术；②术前血常规、血生化、凝血，纠正贫血、电解质紊乱、营养不良、出凝血障碍等，待好转后可考虑手术；③术前胸部影像学、心电图；④术前可选择纤维支气管镜、经支气管镜腔内超声（endobronchial ultrasonography, EBUS）等检查；⑤肺功能检查评估呼吸功能；⑥对基线异常检验检查进行必要的复查；⑦由资深胸外科专家评估手术指征，必要时进行胸外科主导的多学科会诊。

#### 1.2.2 术后评估

① 评估患者神志、呼吸及循环状态；②评估伤口愈合情况；③评估引流管通常情况；④评估有无排痰困难、皮下气肿、肺内啰音、呼吸音不对称、心律失常等手术并发症；⑤术后评估血常规、血生化、凝血，并根据患者术后情况增加必要的检验检查项目。

#### 1.2.3 常规评估

① 询问患者有无新发症状或原有症状加重，详尽且细致的全身体格检查，评估身高、体重、体力评分[美国东部肿瘤协作组（Eastern Cooperative Oncology Group, ECOG）体能状态（performance status, PS）、卡氏PS（Karnofsky PS, KPS）]，必要时疼痛评分；②每个周期全身治疗前均应进行一般检验以及可疑不良反应的针对性检查（表 4）；③一般术后每3个月进行影像学检查，对原发肿瘤进行复查。

### 1.3 围手术期治疗方案的探索

目前正在探索中的新辅助免疫治疗的Ⅲ期临床研究，包括CheckMate 816、KEYNOTE-671、IMpower030、AEGEAN等，设计了ICIs联合化疗的新辅助方案，这也是前期Ⅰ期/Ⅱ期临床试验中，MPR/pCR率最高的方案。因此，对于身体状况良好（PS评分0分-1分）的可手术患者，新辅助ICIs联合化疗应作为优先推荐。除此之外，在众多其他新辅助治疗方案之中，ICIs单药也是重要选择方案之一，对于PD-L1高表达的可手术NSCLC，可考虑行ICIs单药新辅助治疗，但切实疗效仍有待大型Ⅲ期临床试验的结果。另外，PD-1/L1抑制剂+CTLA-4抑制剂的双免疫治疗，尚需大样本研究证实其在围手术期治疗中的安全性。关于新辅助免疫治疗的最佳疗程目前尚无结论，当前的临床研究大多经验性地选择在术前进行2个-4个周期的新辅助治疗。

目前关于NSCLC围手术期免疫治疗的Ⅲ期临床研究，更热衷于在术前完成3个-4个周期的化疗联合免疫新辅助治疗，术后进行免疫单药联合/不联合化疗的辅助治疗。对于术前未经治疗，直接手术的Ⅰb期-Ⅲa期NSCLC来说，目前的Ⅲ期临床研究很少采用化疗联合免疫的辅助治疗的方案，而是更倾向于采用序贯治疗，比如IMpower010和ALCHEMIST研究，均设计了标准术后辅助化疗4个周期之后序贯免疫单药维持治疗组。然而考虑到围手术期化疗对于术后5年生存率的改善仅有5%，效果甚微，因此ANVIL、PEARLS、BR.31这三项研究，均将术后辅助化疗设计为可选项，而免疫单药维持治疗的时间均设计为1年。

总而言之，围手术期免疫治疗的方案选择，暂无高级别的循证医学证据支持。前期结果表明，新辅助免疫联合化疗取得了较好的病理缓解率，但高MPR率/pCR率能否转化为生存获益，尚需等待Ⅲ期临床试验的研究结果证实。而对于术后辅助治疗来说，生存指标是最重要的评价标准，而目前的术后辅助研究大多需2024年以后才能完成，在得到结论之前，无论是化疗联合ICIs、化疗序贯ICIs、或是单药/双药ICIs，都值得尝试和探索。

### 1.4 围手术期irAE

#### 1.4.1 术前irAE

新辅助免疫治疗多在2个-4个周期完成，一些小样本的Ⅱ期临床研究，重点探索了免疫治疗对于外科手术的影响。LCMC3研究^[28]^初步汇报了101例早期可切除NSCLC患者，术前2个周期Atezolizumab之后，3级-4级不良反应发生率为29%，主要为疲劳、发热、食欲减退、转氨酶升高、恶心、关节痛、流感样症状、腹泻、肺炎、贫血等，但患者总体耐受良好，未出现手术延迟。NEOSTAR研究^[17]^评估了Nivolumab单药对比Nivolumab联合Ipilimumab双药免疫新辅助治疗，两组之间不良反应发生率无显著差异，3级-5级不良反应的发生率：高镁血症为4%、低氧血症为4%、严重腹泻为4%、低钠血症为4%，其中单药免疫组有1例患者由于严重不良反应未接受手术，而双药免疫组有4例患者最终未接受手术。

#### 1.4.2 术中中转开胸率

一项评估20例Nivolumab单药新辅助免疫治疗可切除NSCLC（Ⅰa期-Ⅲa期）患者安全性的研究^[31]^显示，13例术前预计接受微创治疗（胸腔镜或机器人手术）患者新辅助免疫治疗后，7例（53.8%）最终因肺门炎症或纤维化转为开胸手术，其中Ⅰa期患者转换率为25%（1/4），Ⅰb期-Ⅲa期患者转换率高达67%（6/9）。手术时间（228 min）、术中出血量（100 mL）与新辅助化疗无显著差异。

#### 1.4.3 术后irAE及并发症

一项纳入20例Nivolumab新辅助免疫单药治疗NSCLC的研究中^[31]^，术后房性心律失常发生率约30%（6/20），心肌梗死为5%（1/20），肺部感染为5%（1/20），肺栓塞为5%（1/20），脓胸为5%（1/20）。NEOSTAR研究显示^[28]^，术前单药Nivolumab治疗2个周期，术后并发症的发生率：持续肺漏气为22%、支气管胸膜瘘为9%、脓胸为4%、肺部感染为4%、非特异性肺炎为4%。NADIM研究^[6]^最新汇报的结果显示，术前Nivolumab联合卡铂紫杉醇方案化疗3个周期后，术后并发症发生率为17.1%（7/41），包括心律失常、持续肺漏气、呼吸道感染、术后疼痛、喉返神经麻痹、血小板减低、术后肺部感染、下肢蜂窝织炎、房颤。

总体而言，可手术NSCLC患者的新辅助免疫治疗相对安全，免疫单药治疗所有级别和≥3级AE发生率分别为23%-57%和4.5%-13%。但目前新辅助免疫治疗几乎均为Ⅰ期/Ⅱ期小样本探索性研究，随访时间较短，数据不完整，对于新辅助免疫治疗相关irAE无法窥其全貌，仍需待更多大规模、前瞻性、长期随访研究结果披露。既往的经验和数据告诉我们，在晚期肿瘤患者中，多种irAE类型均有出现的可能，影响患者预后；而对于可手术的肺癌患者，围手术期irAE也必然会对其后续治疗产生深远影响。因此，良好而规范的围手术期免疫不良反应管理，不仅能够保证整体治疗方案的顺利实施，也会对患者临床结局的改善起到积极正向的作用，广大的肺癌从业临床医生应给予足够的重视。

## 2 irAE治疗

### 2.1 irAE分级

免疫检查点抑制剂对T细胞功能的激活会导致一系列炎症性不良事件的发生，对其确切的病理生理学机制尚未完全了解，目前认为irAE可能通过自身反应性T细胞，自身抗体和细胞因子等多种途径产生。irAE涉及全身多个系统及器官，具体分级根据受累靶器官不同而有所差异，一般而言，1级-2级不良反应无需住院治疗，患者无症状或症状轻微；3级不良反应需住院治疗，患者出现显著症状或症状持续加重；4级不良反应需考虑收入重症监护病房治疗，患者出现威胁生命的症状或体征（表 5）。

**5:** irAE分级 irAE classification

所属器官或系统	疾病名称	分级
注射反应		· G1：轻度暂时性反应, 无需暂停注射，无需特殊治疗 · G2：暂停输液，立即给与系统性治疗（抗组胺药物、NSAIDs、阿片类药物、静脉补液），药物治疗≤24 h · G3：初始治疗后症状延长或反复发作（治疗后症状五显著改善或暂停输液后症状仍反复）住院治疗；需要其他医疗干预措施 · G4：威胁生命，需要紧急干预 · G5：死亡
细胞因子释放综合征		· G1：T≥38 °C，无低血压，低氧血症 · G2：T≥38 °C，低血压（无需血管活性药物），和/或低氧血症（鼻导管≤6 L/min） · G3：T≥38 °C，低血压，需1种血管活性药物，和/或低氧血症（高流量），面罩、Venturi面罩 · G4：T≥38 °C，低血压，需多种血管活性药物，和/或低氧血症（有创或无创机械通气）
皮肤	斑丘疹	· G1：皮损 < 10%体表面积，无症状 · G2：皮损在10%-30% BSA之间，有或无（瘙痒/灼热/紧绷） · G3-G4：皮损 > 30%，有或无（瘙痒/灼热/紧绷）
	瘙痒	· G1：瘙痒感局限且较轻 · G2：瘙痒感强烈，范围广泛，间断性，随着搔抓皮疹发生变化（如水肿、丘疹、皮肤脱落、渗出/结皮） · G3：强烈、广泛、持续性瘙痒；影响ADL及睡眠
	大疱性类天疱疮	· G1无症状，水疱覆盖10%BSA · G2水疱10%-30%BSA，痛性水疱，限制ADL · G3水疱 > 30%BSA，引起水、电解质紊乱，有重症加强护理病房或烧伤病房指征
	苔藓样皮炎	· G1：皮损 < 10%体表面积，无症状 · G2：皮损在10%-30% BSA之间，有或无（瘙痒/灼热/紧绷） · G3-G4：皮损 > 30%，有或无（瘙痒/灼热/紧绷）
	银屑病	· G1：皮损 < 10%体表面积，无症状 · G2：皮损在10%-30% BSA之间，有或无（瘙痒/灼热/紧绷） · G3-G4：皮损 > 30%，有或无（瘙痒/灼热/紧绷）症状
	白癜风	· G1：皮损 < 10%体表面积，无症状 · G2：皮损在10%-30% BSA之间，有或无（瘙痒/灼热/紧绷） · G3-G4：皮损 > 30%，有或无（瘙痒/灼热/紧绷）症状
	皮肤毛细血管增生症	· G1：单个最大直径≤10 mm，伴或不伴破溃出血 · G2：单个最大直径 > 10 mm，伴或不伴破溃出血 · G3：呈泛发性，可以并发皮肤感染，可能需要住院治疗 · G4：多发和泛发，威胁生命 · G5：死亡
	SJS/TEN	· G4
	DRESS	· G4
	急性发热性嗜中性皮病	· G4
呼吸系统	肺炎	· G1：无症状；病变局限于一叶肺或病变范围 < 25%的肺实质 · G2：出现新的呼吸道症状或原有症状加重，包括气短/咳嗽/胸痛/发热，以及所需吸氧条件升高 · G3：症状严重，病变累及所有肺叶或 > 50%肺实质，日常活动受限 · G4：危及生命的呼吸损害
消化系统	腹泻	· G1：小于4次 · G2：4次-6次 · G3-G4： > 6次，结肠炎症状，血红蛋白不稳定，缺血性肠病，穿孔，中毒性巨结肠
	结肠炎	· G1：不超过4次/d，无全身中毒体征，ESR正常 · G2： > 4次/d，轻度贫血，不严重腹痛，低热 · G3-G4：≥6次/d，伴重度肠绞痛，全身中毒症状（T≥37.5 °C，HR≥90 bpm，HGB < 10.5 g），ESR≥30 mm/h，体重迅速下降
	转氨酶升高	· G1： < 3 ULN · G2：3-5 ULN · G3： > 5-20 ULN · G4： > 20 ULN
	高胆红素血症	· 转氨酶 > 3 ULN · 胆红素 > 1.5 ULN
	高淀粉酶血症	· G1：AMY≤3 ULN+LIP≤3 ULN · G2：AMY/LIP 3-5 ULN · G3-G4：AMY/LIP > 5 ULN
	急性胰腺炎	· G1：症状或酶学或影像学其一符合胰腺炎 · G2：症状/酶学/影像学其中两者符合胰腺炎 · G3-G4：症状±酶学±影像学或呕吐或血色素不稳定
循环系统	心包炎	· G1：无症状，心脏标志物检测值或心电图异常 · G2：轻度症状，心脏标志物检测值和心电图异常 · G3：心脏彩超提示左室射血分数 < 50%或局部室壁运动异常；心脏MRI诊断或怀疑心肌炎 · G4：危及生命的心脏异常如恶性心律失常、心源性休克
	心肌炎	
	心肌病	
	心律失常	
	心肌缺血	
内分泌系统	亚临床甲减	· TSH升高（4 mIU/L-10 mIU/L），游离T4正常 · TSH升高（> 10 mIU/L），游离T4正常 · TSH正常或降低，游离T4降低，考虑中枢性甲减
	甲状腺功能减低	· G1：TSH < 10 mIU/L，无症状；只需临床或诊断性观察；暂无需治疗 · G2：持续性TSH > 10 mIU/L，有症状；影响使用工具性日常生活活动 · G3：症状严重；个人自理能力受限；需要住院治疗 · G4：危及生命；需要紧急干预处理
	甲状腺毒症	· TSH降低，游离T4正常或升高
	中枢性甲减	· TSH低或抑制伴有不适当的游离T4低
	垂体炎	· G1：无症状或轻度症状 · G2：中度症状，能够进行日常生活活动 · G3-G4：重度症状，医学上有重要意义或危及生命；自理性日常生活活动受限
	孤立性肾上腺功能减退症	· G1：无症状或轻度症状 · G2：中度症状，能够进行日常生活活动 · G3-G4：重度症状，医学上有重要意义或危及生命；自理性日常生活活动受限
神经系统	重症肌无力	· G1：无症状（任何涉及颅内神经病变均为G2） · G2：限制ADL评分 · G3-G4：限制自理能力，限制行走，任何程度的吞咽困难、面部无力、呼吸机无力或快速进展性症状限制行走或呼吸
	格林巴利综合征	
	周围神经病	
	炎性肌病	
	无菌性脑膜炎	
	脑炎	
	多发性硬化	
	视神经炎	
	横断性脊髓炎	
	面神经麻痹	
风湿免疫系统	炎性关节炎	· G1：轻度疼痛，伴有炎症、红斑或关节肿胀 · G2：中度疼痛伴炎症、红斑或关节肿胀的迹象，影响使用工具性日常生活活动 · G3-G4：伴有炎症、红斑或关节肿胀迹象的剧烈疼痛；不可逆转的关节损伤；致残；个人自理能力受限
	肌炎	· G1：轻度症状伴或不伴疼痛 · G2：中度症状伴或不伴疼痛，影响使用工具性日常生活活动 · G3-G4：严重症状伴或不伴疼痛，个人自理能力受限
	风湿性多肌痛	· 轻度：轻度疼痛和/或僵硬，日常生活活动不受限 · 中/重度：影响工具性日常生活活动或自理能力的疼痛和/或僵硬
	巨细胞动脉炎	· 视力改变、头痛、头皮压痛、下颌跛行
血液系统	自身免疫性溶血性贫血	· G1：Hgb < LLN-100 g/L · G2：Hgb < 100 g/L-80 g/L · G3：Hgb < 80 g/L · G4：危及生命，需要紧急干预治疗
	免疫性血小板减少	· G1：血小板 < 100/*μ*L · G2：血小板 < 75/*μ*L · G3：血小板 < 50/*μ*L · G4：血小板 < 25/*μ*L
	AA	· 中度AA：①骨髓细胞增生程度 < 30%；②无严重全血细胞减少；③三系血液成分中至少两系低于正常 · SAA：①骨髓细胞增生程度 < 25%或②骨髓活检显示细胞增生程度小于50%，其中造血细胞少于30%，并且存在①网织红细胞计数 < 40, 000/*μ*L；中性粒细胞 < 500/*μ*L；PLT < 20, 000/*μ*L · 极重度AA：符合SAA标准且ANC < 200/*μ*L
肾脏	肾炎	· G1：肌酐 > 0.3 mg/dL；肌酐升高至1.5倍-2倍基线水平 · G2：肌酐水平升高至2倍-3倍基线水平 · G3：肌酐水平 > 4.0 mg/dL；肌酐升高至 > 3倍基线水平 · G4：威胁生命；需要替代治疗
眼	葡萄膜炎	· G1：症状轻微 · G2：前葡萄膜炎 · G3：后葡萄膜炎或全葡萄膜炎 · G4：视力20/200（法律意义上失明）
	Vogt-Koyanagi-Harada综合征样改变	· G1：症状轻微 · G2：视力20/40或更好 · G3：视力弱于20/40 · G4：视力20/200（法律意义上失明）
BSA：牛血清白蛋白；NSAIDs：非甾体类抗炎药；SJS：Stevens-Johnson综合征；TEN：中毒性表皮坏死松解症；DRESS：药疹伴嗜酸粒细胞增多和系统症状；ADL：日常生活活动；LLN：正常值下限；AA：再生障碍贫血；T：体温；HR：心率；HGB：血红蛋白；ESR：红细胞沉降率		

### 2.2 一般原则

① 坚持以“预防、评估、检查、治疗、监测”作为免疫检查点抑制剂安全管理的重要原则，做到早期发现、准确诊断、精准治疗；②鼓励与特定疾病的专科医生密切协商；复杂病例或多系统irAE可能需要转诊至三级医疗机构进行诊治，对于危重症irAE需争分夺秒，避免延误最佳治疗时机；③出现≥2级irAE应暂停ICIs治疗，若症状或/和实验室检验降至1级及以下可恢复治疗；若症状持续 > 1周，应开始糖皮质激素（glucocorticoid, GC）治疗；④出现3级-4级irAE患者，应给与GC治疗，症状逐步恢复至1级及以下后开始减量，总体疗程一般维持在4周-6周；⑤对于出现4级irAE（非替代治疗可控制的内分泌irAE）患者，需永久停用ICIs治疗；对于≥2级irAE持续6周以上、GC无法在12周内减量至泼尼松10 mg以下的患者也许考虑永久停用ICIs治疗；⑥若静脉GC≥3 d（72 h）症状无改善患者，应考虑免疫调节剂或其他方案治疗；⑦在ICIs治疗过程中，允许使用灭活或灭活制剂的疫苗，但不建议在ICIs治疗期间接种活疫苗。

### 2.3 预防原则

① 在大剂量GC（1 mg/kg/d-2 mg/kg/d），尤其是冲击量GC期间或者合并消化道出血高危因素的患者，考虑加用质子泵抑制剂或H2受体阻滞剂；②对于泼尼松≥20 mg/d，持续4周或4周以上者，需考虑预防卡氏肺孢子虫肺炎；对更长时间使用GC（泼尼松 > 20 mg/d，持续6周-8周以上）的患者，可考虑使用抗真菌药物预防真菌感染；③长期使用GC的患者有发生骨质疏松症的风险，推荐口服补充维生素D和钙剂，并监测骨代谢指标，必要时考虑加用抗骨质疏松药物治疗；④GC使用期间，应注意患者宣教，避免人多密集场所或避免接触感染源；加强个人防护（戴口罩、勤洗手等）；注意饮食卫生及食量控制，避免大幅度体重增加；监测血糖、血压、电解质等。

### 2.4 GC使用原则

GC是由肾上腺皮质分泌的甾体类激素，对机体的发育、生长、代谢以及免疫功能等方面起着重要调节作用，具有抗炎、抗过敏、抗休克、免疫调节等功能。通常认为，GC通过经典的基因组效应（转录机制）发挥作用，即脂溶性GC可以自由或经转运蛋白透过细胞膜进入细胞内，与胞浆内糖皮质激素受体α结合，导致胞浆内糖皮质激素受体α构象改变，与热休克蛋白90（heat shock protein 90, HSP90）、HSP70、HSP56、HSP40等分子伴侣解离。解离后的分子伴侣与靶基因特异序列激素反应原件（glucocorticoid response elements, GRE）或负性糖皮质激素反应原件（negative glucocorticoid response elements, nGRE）相结合，影响细胞核的转录和翻译过程，上调免疫蛋白的合成，下调炎症介质的释放，从而完成抗炎、免疫调节。因基因转录和蛋白质合成需要一定的时间，因此经典的基因组效应需要数小时或数天产生显著临床作用。GC的非基因组效应（非转录机制）可与特异性的糖皮质激素受体作用和（或）与细胞膜的物理化学作用和（或）与细胞浆中经典糖皮质激素受体的特异性作用，最终达到快速（几秒钟或数分钟）起效的生理或药理作用。因而，不同种类的GC具有不同强度和不同速度的效力及毒副作用（表 6）。根据GC的药代动力学特征，按照半衰期长短，GC可分为短效、中效及长效（表 6）。大多数口服GC 30 min即可吸收，口服生物利用度较高。

**6:** 激素种类及其药代动力学特征 Hormone species and their pharmacokinetic characteristics

剂量等级	短效激素（作用时间8 h-12 h）			中效激素（作用时间18 h-36 h）				长效激素（作用时间36 h-54 h）		基因组效应	非基因组效应
	可的松	氢化可的松		泼尼松	泼尼松龙	甲基泼尼松龙		地塞米松	倍他米松		
等效剂量	25 mg	20 mg		5 mg	5 mg	4 mg		0.75 mg	0.6 mg		
低剂量	≤37.5 mg	≤30 mg		≤7.5 mg	≤7.5 mg	≤6 mg		≤1.125 mg	≤0.9 mg	+	
中等剂量	37.5 mg-150 mg	30 mg-120 mg		7.5 mg-30 mg	7.5 mg-30 mg	6 mg-24 mg		1.125 mg-6 mg	0.9 mg-3.6 mg	++	+
大剂量	150 mg-500 mg	120 mg-400 mg		30 mg-100 mg	30 mg-100 mg	24 mg-80 mg		6 mg-15 mg	3.6 mg-12 mg	++	+
超大剂量	> 500 mg	> 400 mg		> 100 mg	> 100 mg	> 80 mg		> 15 mg	> 12 mg	+++	++
冲击剂量				≥250 mg	≥250 mg	> 200 mg		> 37.5 mg		+++	+++

临床上irAE治疗很大程度上依赖于早期使用GC，目前国内外各大指南对于GC剂量及疗程意见大致相同，个别存在细节差异，具体可参考表 7。总体而言，对于1级irAE不推荐应用GC或免疫调节剂，无需暂停ICIs；对于2级irAE需暂停ICIs，建议局部或全身中等量中效GC（0.5 mg/kg/d-1 mg/kg/d）；3级-4级irAE需永久停用ICIs，建议全身大剂量乃至冲击剂量中效GC，可在GC应用后3 d-5 d内根据症状加用免疫调节剂或其他治疗手段。

**7:** NCCN指南、ESMO指南、SITC指南、CSCO指南中irAE治疗糖皮质激素起始剂量建议 Suggestions on initial dose of glucocorticoid for irAE treatment in NCCN guidelines, ESMO guidelines, SITC guidelines and CSCO guidelines

irAE	级别	NCCN（2020.V1）	ESMO（2017）	SITC（2017）	ASCO（2018）	CSCO（2019）
注射反应	2级-4级	未建议应用	/	可考虑应用	/	未建议应用
皮肤毒性	2级	Pred 0.5 mg/kg/d-1 mg/kg/d	/	Pred 0.5 mg/kg/d-1 mg/kg/d	Pred 1 mg/kg/d（或等效剂量）	Pred 0.5 mg/kg/d-1 mg/kg/d
	3级-4级	皮疹：pred 0.5 mg/kg/d-1 mg/kg/d 大疱性皮炎：pred 1 mg/kg/d-2 mg/kg/d SJS/TEN：pred 1 mg/kg/d-2 mg/kg/d	3级：泼尼松0.5 mg/kg/d-1 mg/kg/d 4级：MP 1 mg/kg/d-2 mg/kg/d	pred 1 mg/kg/d-2 mg/kg/d	Pred 1 mg/kg/d-2 mg/kg/d（或等效剂量）	pred 0.5 mg/kg/d-1 mg/kg/d
肺毒性	2级	Pred 1 mg/kg/d-2 mg/kg/d	Pred 1 mg/kg/d	MP 1 mg/kg/d	Pred 1 mg/kg/d-2 mg/kg/d	MP 1 mg/kg/d-2 mg/kg/d
	3级-4级	MP 1 mg/kg/d-2 mg/kg/d	MP 2 mg/kg/d-4 mg/kg/d	MP 2 mg/kg/d	MP 1 g/d，5 d	MP 2 mg/kg/d
胃肠道毒性 （腹泻/结肠炎）	2级	Pred 1 mg/kg/d-2 mg/kg/d	Pred 0.5 mg/kg/d-1 mg/kg/d	Pred 1 mg/kg/d	Pred 1 mg/kg/d	Pred 1 mg/kg/d
	3级-4级	MP 1 mg/kg/d-2 mg/kg/d	MP1 mg/kg/d-2 mg/kg/d	MP 1-2 mg/kg/d	Pred 1 mg/kg/d-2 mg/kg/d	MP 2 mg/kg/d
肝毒性	2级	Pred 0.5 mg/kg/d-1 mg/kg/d	Pred 1 mg/kg/d	Pred 0.5 mg/kg/d-1 mg/kg/d	Pred 0.5 mg/kg/d-1 mg/kg/d	Pred 0.5 mg/kg/d-1 mg/kg/d
	3级-4级	3级：pred 1-2 mg/kg/d 4级：pred 2 mg/kg/d	3级：胆红素/INR/白蛋白正常MP 1 mg/kg/d；胆红素升高/INR升高/白蛋白下降MP 2 mg/kg/d 4级：MP 2 mg/kg/d	Pred 1 mg/kg/d-2 mg/kg/d	3级：pred 1 mg/kg/d-2 mg/kg/d 4级：pred 2 mg/kg/d	MP 1 mg/kg/d-2 mg/kg/d
心脏毒性	2级	/	/	可疑心肌炎建议应用大剂量激素（case-by-case）	Pred 1 mg/kg/d-2 mg/kg/d	/
	3级-4级	MP 1 g/d，3 d-5 d	/	MP 1 mg/kg/d	MP 1 g/d	MP 1g/d，3 d-5 d
垂体炎	1级	Pred 1 mg/kg/d-2 mg/kg/d	/	/	HCT 15 mg/d-30 mg/d	Pred 1 mg/kg/d-2 mg/kg/d
	2级		Pred 0.5 mg/kg/d-1 mg/kg/d	HCT 10 mg/m^2^	Pred 20 mg/d或HCT 30 mg/d-50 mg/d	
	3级-4级		MP 1 mg/kg/d	Pred 1 mg/kg/d	Pred 1 mg/kg/d-2 mg/kg/d	
关节炎	2级	Pred 0.5 mg/kg/d	Pred 10 mg-20 mg		Pred ≤10 mg/d	Pred 0.5 mg/kg/d
	3级-4级	pred 1 mg/kg/d	MP 0.5 mg/kg/d-1 mg/kg/d		Pred 0.5 mg/kg/d-1 mg/kg/d	Pred 1 mg/kg/d
神经毒性	2级	MG：pred 20 mg/d GBS：MP 1 g/d，5 d 周围神经病：pred 0.5 mg/kg/d-1 mg/kg/d 无菌性脑膜炎：pred 0.5 mg/kg/d-1 mg/kg/d 脑炎：MP 1 mg/d-2 mg/d	GBS：MP 1 mg/kg/d-2 mg/kg/d 周围神经病：pred 0.5 mg/kg/d-1 mg/kg/d 无菌性脑膜炎：pred 0.5 mg/kg/d-1 mg/kg/d	MP 0.5 mg/kg/d-1.0 mg/kg/d	MG：pred 1 mg/kg/d-1.5 mg/kg/d；GBS：MP 2 mg/kg/d-4 mg/kg/d； 周围神经病：pred 0.5 mg/kg/d-1 mg/kg/d 自主神经病：pred 0.5 mg/kg/d-1 mg/kg/d 无菌性脑膜炎：pred 0.5 mg/kg/d-1 mg/kg/d 脑炎：MP 1 mg/d-2 mg/d	MG：pred 1 mg/kg/d-1.5 mg/kg/d； GBS：甲基泼尼松2 mg/kg/d-4 mg/kg/d； 无菌性脑膜炎：1 mg/kg/d 脑炎：甲基泼尼松1 mg/kg/d-2 mg/kg/d
	3级-4级	MG：MP 1 mg/kg/d-2 mg/kg/d GBS：MP 1 g/d，5 d 周围神经病：MP 1 g/d，5 d 无菌性脑膜炎：pred 1 mg/kg/d-2 mg/kg/d 脑炎：MP 1 g/d，3 d-5 d 横断性脊髓炎：MP 1 g/d，3 d-5 d	周围神经病：pred 2 mg/kg/d 无菌性脑膜炎：MP 1 mg/kg/d-2 mg/kg/d 横断性脊髓炎：MP 2 mg/kg/d（可考虑1 g/d）	MP 1 mg/kg/d-2 mg/kg/d	MG：MP 1 mg/kg/d-2 mg/kg/d； GBS：MP 2 mg/kg/d-4 mg/kg/d； 周围神经病：MP 2 mg/kg/d-4 mg/kg/d 自主神经病：MP 1 g/d，3 d 无菌性脑膜炎：MP 1 g/d 脑炎：MP 1 g/d，3 d-5 d； 横断性脊髓炎：MP 1 g/d，3 d-5 d	MG：MP 1 mg/kg/d-2 mg/kg/d； GBS：甲基泼尼松2 mg/kg/d-4 mg/kg/d； 无菌性脑膜炎：甲强龙1 mg/kg/d 脑炎：甲强龙1 mg/kg/d-2 mg/kg/d；横断性脊髓炎：1 g/d，3 d-5 d
贫血	2级	/	/	/	Pred 0.5 mg/kg/d-1 mg/kg/d	AIHA：pred 0.5 mg/kg/d-1 mg/kg/d AA：未建议应用激素
	3级-4级	/	/	/	Pred 1 mg/kg/d-2 mg/kg/d	AIHA：1 mg/kg/d-2 mg/kg/d AA：未建议应用
血小板减少	2级	/	/	/	Pred 1 mg/kg/d	Pred 0.5 mg/kg/d-2 mg/kg/d
	3级-4级	/	/	/	Pred 1 mg/kg/d-2 mg/kg/d	Pred 1 mg/kg/d-2 mg/kg/d
肾毒性	2级	Pred 0.5 mg/kg/d-1 mg/kg/d	Pred 0.5 mg/kg/d-1 mg/kg/d	开始使用，剂量个体化制定	Pred 0.5 mg/kg/d	Pred 0.5 mg/kg/d-1 mg/kg/d
	3级-4级	Pred 1 mg/kg/d-2 mg/kg/d	MP 1 mg/kg/d-2 mg/kg/d		Pred 1 mg/kg/d-2 mg/kg/d	MP 1 mg/kg/d-2 mg/kg/d
眼毒性	3级-4级	全身糖皮质激素（眼科专科意见）		全身及局部糖皮质激素（眼科专科意见）	MP 1 mg/kg/d-2 mg/kg/d	全身及局部糖皮质激素（眼科专科意见）
NCCN：美国国立综合癌症网络；ESMO：欧洲肿瘤内科学会；SITC：美国肿瘤免疫治疗学会；ASCO：美国临床肿瘤学会；CSCO：中国临床肿瘤学会；AIHA：自身免疫性溶血性贫血；AA：再生障碍性贫血；MG：重症肌无力；GBS：格林巴利综合征；pred：泼尼松；MP：甲基泼尼松龙；HCT：氢化可的松						

### 2.5 静注免疫球蛋白（intravenous immunoglobulin, IVIG）

IVIG来自于正常人血浆，是一类由二硫键连接四条肽链组成的结构对称的具有抗体活性的球蛋白，其中两条长链称为重链（heavy chain，H链），两条短链称为轻链（light chain，L链）；重链分为*μ*（IgM）、γ（IgG）、α（IgA）、δ（IgD）、ε（IgE），轻链分为κ型和λ型。IVIG通过抑制致病性自身抗体的产生、抑制补体产生、抑制Fc受体、下调致病性细胞因子和黏附分子、抑制T细胞功能等多种机制，达到免疫调节作用，被广泛用于各种重症irAE的治疗，包括格林巴利综合征、重症肌无力、大疱性皮疹、SJS/TEN、DRESS、血小板减少等抗体介导或伴炎症反应的irAE。

初次应用，总剂量为2 g/kg，可3 d-5 d内分次给予，目前临床多采用400 mg/kg/d，连用3 d-5 d为1个疗程，若疾病复发，可重复此疗程。IVIG需缓慢静脉输注，开始为1 mL/min，最快不超过3 mL/min；应单独应用，不应和其他药品混合滴注；药品开启后，应一次性输注完毕，不得分次或给予第二人输注。

IVIG是一种相对安全的治疗药物，副作用发生率为1%，常见副作用包括头痛、脊背疼痛、恶心、呕吐、腹泻、面部潮红、发热、寒战、呼吸急促、胸闷、低血压、高血压及皮疹。不良反应多为暂时性，通常发生在第一次或第二次输注时，且与静滴速度过快、使用不同厂家制剂相关。减慢输注速度反应可减轻，极少情况下需在输注前30 min给与小剂量GC或抗组胺药物。因IVIG中含有少量IgA，先天性IgA缺乏症患者易出现过敏反应，需严格慎用/禁用。

### 2.6 其他治疗药物/方法

因对于围手术期患者，长期中等剂量及以上剂量GC可能增加感染或延迟愈合，经多学科讨论后，对于重症irAE可早期采用多种手段联合治疗。

#### 2.6.1 肿瘤坏死因子α（tumor necrosis factor-α, TNF-α）抑制剂

① 48 h-72 h内对GC治疗无反应的重症irAE，可早期（72 h内）开始TNF-α抑制剂治疗（如英夫利昔单抗5 mg/kg；阿达木单抗、依那西普也有个案报道）；②需要对接受GC和英夫利昔单抗治疗的患者进行密切监测和随访，以评估疗效。根据需要可考虑2周、6周后重复给药；③英夫利昔单抗有再次激活乙型肝炎病毒的潜在风险，因此在接受TNF-α抑制剂治疗前需要检测乙型和丙型肝炎病毒，且对于HBV/HCV携带者需要在治疗期间和治疗结束后数月动态监测；④英夫利昔单抗有激活结核病（tuberculosis, TB）的风险。在开始TNF抑制剂治疗前需检测潜伏性/活动性TB；在紧急情况下需要给予抗TNF-α抑制剂治疗时，无需等待TB的检测结果。

#### 2.6.2 白细胞介素6受体（interleukin-6 receptor, IL-6R）抑制剂

① 48 h-72 h内对GC治疗无反应的严重irAE患者，评估存在炎症状态后，可早期（72 h内）开始IL-6R抑制剂治疗（如托珠单抗4 mg/kg-8 mg/kg）；根据需要可考虑8 h后重复应用，24 h不可超过3次；②慢性或复发性感染患者需慎用，对于存在结核感染、侵袭性真菌感染、细菌感染、病毒感染和其他机会性感染患者，需在应用托珠单抗前进行充分抗感染治疗；应用过程中密切监测患者血液学指标、肝肾功能。

#### 2.6.3 抗CD20（cluster of differentiation 20）单抗

① CD20是一种由MS4A1基因编码位于B淋巴细胞表面的跨膜磷脂蛋白。CD20作为B细胞的表面抗原，可标记前B细胞到成熟B细胞阶段，在造血干细胞、祖B细胞及成熟浆细胞表面不表达；②以CD20为靶点的单克隆抗体杀伤肿瘤机制主要包括：①抗体依赖的细胞毒作用（antibody dependent cell mediated cytotoxicity, ADCC）、补体依赖的细胞毒作用（complement dependent cytotoxicity, CDC）以及单克隆抗体与CD20分子结合引起的直接效应，包括抑制细胞生长、改变细胞周期以及凋亡；③目前NCCN指南推荐利妥昔单抗用于治疗GC耐药大疱性皮炎、难治性神经损害（重症肌无力、无菌性脑膜炎、脑炎）；④用法：1)大疱性皮炎：利妥昔单抗1, 000 mg，*Q2w*，4 wk→500 mg，12月/次或18月/次；2)神经损害：利妥昔单抗375 mg/m^2^，*Qw*，4 wk或500 mg/m^2^，*Q2w*，4 wk。

#### 2.6.4 霉酚酸酯（mycophenolate mofetil, MMF）

① MMF口服后在体内抑制次黄嘌呤核苷磷酸脱氢酶，使鸟嘌呤核苷酸合成减少，因而能选择性抑制T、B淋巴细胞的增殖和功能；②对于难治性肝炎、肺炎、大疱性疾病的irAE患者可考虑应用1 g/d-2 g/d，分2次口服，后续可根据患者症状变化及专科医师会诊意见调量；③可出现胃肠道反应，药物减量或停用后可明显缓解；具有致畸作用；长期应用可继发机会性感染。

#### 2.6.5 环孢素A（cyclosporin A, CsA）

① 环孢素是一种从真菌代谢产物中提取的含有11个氨基酸的环状多肽，其与细胞内免疫嗜素亲环蛋白结合，抑制Th细胞活化，IL-2的反应性，进一步影响B淋巴细胞分化，抑制由其介导的免疫反应；②目前指南建议用于治疗ICIs引起的再生障碍性贫血、难治性肾损害、难治性神经损害；③初始剂量4 mg/kg/d-5 mg/kg/d，分2次口服（*Q12h*），起效后缓慢减量至2 mg/kg/d-3 mg/kg/d；对于血清肌酐升高的患者，初始剂量为2.5 mg/kg/d；使用过程中血清肌酐较基础值升高30%，则应考虑减量（0.5 mg/kg/d-1.0 mg/kg/d）。监测血药谷浓度，安全窗100 ng/mL-200 ng/mL；④环孢素可引起肾小管间质及肾血管的结构和功能改变，导致肾间质纤维化、血管透明样变性、肾小球硬化等肾毒性；其中急性肾毒性多于血流动力学下降相关，减量或停药后可缓慢恢复；⑤对于存在水痘、带状疱疹等病毒感染患者需禁用。

#### 2.6.6 血浆置换

① 血浆中存在各种分子质量不等的生理物质和致病因子，血浆置换可通过膜式血浆分离或离心式血浆分离的方法，从全血中分离出异常血浆，以清除其中含有的致病因子（如毒性物质、细胞因子、炎症介质等），同时向体内补充等量新鲜血浆或其他替代品，达到暂时减轻和治疗疾病的目的；②血浆置换通过去除细胞免疫和体液免疫的抑制因子，可暂时达到恢复免疫功能的作用，并促进T细胞亚群恢复正常比例。输入的免疫球蛋白分子的Fc段可暂时性封闭单核细胞和巨噬细胞的Fc受体，减少单核细胞和巨噬细胞对结合有特异性抗体的靶细胞的结合和损伤。因此，血浆置换对大多数疾病都并非病因性治疗，只是比药物更迅速、更有效的降低致病因子的浓度，终止由此导致的损害，使疾病得以暂时性缓解；③目前NCCN指南推荐用于重症肌无力、格林巴利综合征、免疫性脑炎、横断性脊髓炎等神经系统疾病，通常作为二线治疗方案。现对于重症或进展迅速的神经系统irAE的治疗成功率参差不齐；④由于体外循环容量过大或回输液胶体渗透压偏低等在体外循环开始1 h之内可能出现低血压；因血浆置换过程中需应用肝素抗凝，体外循环30 min后可能出现穿刺部位渗血、血肿、消化道出血等；体外循环时肝素用量不足或血流不畅、中断或血流量过小或高龄患者，体外循环过程中可能出现血浆分离及管路阻塞等；对肝素、鱼精蛋白过敏患者禁用。

目前irAE治疗中涉及的还包括环磷酰胺、甲氨蝶呤、柳氮磺胺吡啶、来氟米特、艾曲波帕等药物，但多为个案报道，尚未形成共识或诊疗意见，临床治疗过程中对于部分难治性irAE经多学科讨论后给予个体化治疗。

## 3 irAE监测

一旦开启ICIs治疗，需全程考虑irAE可能性，包括治疗中症状及检查检验结果的动态管理、治疗后长时间随访、irAE治疗期间疗效及并发症。

### 3.1 ICIs治疗中监测

在ICIs治疗过程中，需要定期对机体功能状态和脏器功能进行检测，从而早期、及时发现毒性（表 4）。

### 3.2 ICIs治疗后监测

是指ICIs治疗结束一段时间内，定期或不定期对机体功能状态和脏器功能进行检测，从而发现一些迟发性毒性。部分毒性出现时间较晚，尤其是肾功能、垂体功能，目前认为患者在ICIs治疗结束后，至少监测随访1年。

### 3.3 GC及免疫调节剂治疗后监测

是指因irAE应用GC或免疫调节剂治疗后，定期对irAE症状、体征及异常检查检验治疗进行评估和检测，同时包括对GC或免疫调节剂相关副作用及机会性感染的监测。

建议至少每72 h对患者相关irAE症状、体征及检查检验指标进行评估，重新分级，调整治疗方案及决策。对于重症及危重症患者，评估间隔应进一步缩小，24 h或48 h。

应用GC患者，副作用发生风险与GC剂量及疗程呈正相关，低剂量短时间GC副作用风险明显减低。患者第一次使用GC后，即可出现神经精神症状，3 d左右出现水钠潴留、电解质紊乱、心率增快、血压升高等情况，1周内出现血糖升高，3周或更长时间出现机会性感染，8周或更长时间出现真菌感染、骨质疏松等，12周以上可出现Cushing综合征、肾上腺皮质功能抑制等内分泌疾病。使用GC和（或）免疫调节剂期间，严密监测新发感染或潜伏感染的激活（表 8）。

**8:** GC及免疫抑制剂治疗合并感染临床诊断及治疗建议 Clinical diagnosis and treatment suggestions of GC and immunosuppressant in the treatment of complicated infection

感染	临床常用检查检验	临床常用治疗建议
细菌感染	①血常规、炎症指标（ESR、CRP、PCT） ②病原涂片、培养及病原DNA-NGS（痰、尿、便、外周血、导管血、咽拭子） ③影像学确定感染灶 ④肺部感染，可考虑气管镜毛刷以及灌洗液病原涂片、培养、DNA-NGS测序 ⑤其他实体脏器感染，可考虑活检组织送检病原检测	根据院内菌群进行经验性抗感染，后可根据药敏结果选择抗生素
结核分枝杆菌/非结核分支杆菌	①PPD试验 ②外周血：TB-Ab、T-Spot.TB ③痰/气管镜吸取物：抗酸染色、弱抗酸染色、分枝杆菌培养、TB/NTM核酸测定、结核和利福平耐药的快速分子鉴定（X-pert） ④病灶组织：研磨后送检病原 ⑤胸部影像学	TB：抗结核治疗 NTM：根据菌种选择用药
难辨梭菌感染	①便：难辨梭菌毒素测定、难辨梭菌培养 ②必要时可考虑肠镜及活检	甲硝唑*：口服，250 mg，*Tid-Qid* 万古霉素*：口服，125 mg，*Qid* 一般疗程7 d-14 d
真菌感染	①外周血：血常规/G试验/GM试验/血培养 ②痰/气管镜吸取物/气管镜灌洗液：真菌涂片、真菌培养、病原DNA-NGS ③尿培养 ④胸部影像学	经验性抗真菌治疗，后续根据菌属抗真菌治疗
HBV/HCV感染	①乙肝五项、HBV-DNA ②HCV-Ab、HCV-RNA ③肝功能	结合感染科专科意见，抗病毒治疗
HIV感染	HIV-Ab、HIV-RNA、T细胞亚群	结合感染科专科意见，抗病毒治疗
CMV感染	①外周血：CMV-DNA、巨细胞病毒抗原血症（CMVpp65） ②若CMV肠炎，完善肠镜及活检，病理可见CMV包涵体	更昔洛韦*：静脉，5 mg/kg，Q12h*14 d→Qd*7 d（肌酐清除率 > 60 mL/min/kg） 膦甲酸钠*：静脉，60 mg/kg，Q8h*14 d→90 mg/kg，Qd*7 d（肌酐清除率正常）
卡氏肺孢子虫感染	①外周血：血常规（淋巴细胞绝对值）、G试验、LDH ②痰/气管镜吸取物/气管镜灌洗液：六胺银染色、PCP-DNA、病原DNA-NGS ③胸部影像学	复方磺胺甲恶唑*（SMZ 75 mg/kg/d+TMP 15 mg/kg/d），2周-3周；重症患者建议加用GC 40 mg Q12h*5 d→40 mg Qd*5 d→20 mg Qd*11 d （磺胺过敏患者慎用）
*临床常用药物及剂量，需结合患者感染灶、肝肾功能情况及专科意见，酌情进行调整。ESR：血沉；CRP：C反应蛋白；PCT：降钙素原；DNA-NGS：脱氧核糖 核苷酸-二代测序；PPD：结核菌素试验；TB-Ab：结核分枝杆菌抗体；T-Spot.TB：结核感染T细胞斑点试验；LDH：乳酸脱氢酶；GC：糖皮质激素		

## 4 危重及难治性irAE的管理

尽管轻度irAE（1级-2级）和大部分3级-4级的irAE经过早期GC的治疗后可控制良好，但仍有一小部分irAE临床表现严重或不能通过类固醇有效控制，属于危重或难治类型，患者后续可能因irAE未控制、类固醇使用继发的不良反应或原发肿瘤进展等原因危及生命。2018年发表在*JAMA Oncology*期刊上的一篇综述针对致死性irAE的发生情况进行了分析和总结^[32]^，研究共筛选了31, 059例免疫相关不良事件报告，其中613例为致死性irAE。不同治疗药物的致死性irAE的发生类型不同，其中接受Ipilimumab单药治疗的患者中，最常见的致死性irAE为结肠炎/腹泻（70%），而接受抗PD-1/PD-L1抑制剂单药治疗的患者中最常见的为肺炎（35%），其次为肝炎（22%）、神经系统毒性（15%），其他还包括皮肤病等（8%）。另外，免疫联合治疗常见的致死性irAE是结肠炎（37%）和心肌炎（25%）。在所有致死性irAE中，心肌炎的致死率最高（39.7%）。因此，针对这些危重及难治性irAE的相关研究进展进行了总结，以期为临床专家提供参考依据。

### 4.1 免疫相关性肺炎

近来年，随着免疫治疗适应症的扩大，更多复杂的方案使用，使得免疫相关性肺炎的发生率有所增加，且致死率也逐渐升高。Moey等^[33]^对VigⅠbase数据库和全球药品安全性病例报告（individual case safety report, ICSR）数据库中致死性免疫相关肺炎的发生情况进行了汇总显示，2010年-2014年期间致死性免疫相关肺炎的发生率为1例/月，而在2017年-2018年期间已经升高至10例/月，免疫治疗相关肺炎发生的中位时间为2.1个月，且致死性肺炎的发生时间较非致死性肺炎要更早（平均24 d *vs* 53 d；*P* < 0.000, 1）。因此，临床上需重点关注，早期识别，给与及时干预。

对于3级及以上肺炎，推荐收入重症加强护理病房（intensive care unit, ICU）治疗，行支气管镜和支气管肺泡灌洗以评价肺部情况，对于不典型病变可考虑进行活检。对于经验性使用广谱抗生素，予以大剂量静脉类固醇激素治疗[如（甲基）泼尼松龙1 mg/kg/d-4 mg/kg/d或等效药物]，根据患者症状及影像改善情况，逐步减量。10%-15%的患者对激素治疗可能不敏感，建议对皮质类固醇治疗2 d无好转的患者加用免疫抑制剂治疗，可选择英夫利西单抗、霉酚酸酯或环磷酰胺。指南均建议GC减量应该非常缓慢谨慎，4周-8周逐渐减停。建议可静脉注射免疫球蛋白，建议ICU的支持，支气管镜检查指导治疗应该成为常规，可以纠正经验治疗的偏差^[34]^。

### 4.2 免疫相关性心脏毒性

已报道的不良反应包括心肌病变（心肌炎为主）、心包疾病、心律失常、急性冠脉综合征和瓣膜病变等。心脏不良事件具有高致死性，其中心肌炎的致死率高达39.7%-50%，心包疾病的致死率可达21%^[35]^。治疗前应进行详细心血管相关基线检查（表 4），发生严重心脏毒性需立即请心内科会诊，完善心电图检查、心肌损伤标志物（肌酸激酶和肌钙蛋白）、炎性标志物（红细胞沉降率、C反应蛋白、白细胞等）、甲状腺功能，并动态观察心电图、心肌损伤标志物（肌酸激酶和肌钙蛋白）的变化，完成心脏彩超和（或）心肌增强MRI检查。如明确有心肌损害，应给予患者持续心电监护，停用ICIs。如考虑为重症心肌炎，应立即给予甲基泼尼松龙冲击，1 g/d，持续3 d-5 d，NCCN/美国临床肿瘤学会（American Society of Clinical Oncology, ASCO）/CSCO指南均推荐治疗至心功能恢复基线后，在4周-6周内逐渐减量。激素治疗24 h无改善时考虑加用免疫球蛋白、抗胸腺细胞球蛋白（antithymocyte globulin, ATG）、IL-6R抑制剂、英夫利昔单抗（但应注意的是，英夫利昔单抗与心力衰竭有关，对中-重度心力衰竭患者禁用大剂量英夫利昔单抗）等。如出现心包炎，在大剂量GC治疗的基础上，应警惕心包填塞。严重心脏相关毒性反应在给予免疫抑制治疗的同时，需严密心电监护、维持电解质平衡、调整容量负荷，及时调整抗心力衰竭或抗心律失常的药物和器械治疗也尤为重要^[34]^。

### 4.3 免疫相关性神经系统不良反应

免疫相关神经系统的发生率大约为3.8%-12%，其中CTLA-4联合PD-1治疗的发生率最高。与心脏毒性类似，严重神经系统的发生率并不高（1%以下），但其致死率可达15%。严重的神经毒性及其基本的处理原则包括：①格兰巴雷综合症（Gran Barre syndrome, GBS）：2级症状即需永久停止ICIs，住院或入住ICU监护病房，密切监测神经系统症状和呼吸功能，请神内科会诊。可以使用免疫球蛋白，0.4 g/kg/d进行治疗，或者采用血浆置换，连续5 d。对疼痛患者，给予非阿片类药物治疗疼痛；②重症肌无力：3级以上需要永久停止ICIs，住院治疗，甲基泼尼松龙起始量为1 mg/kg/d-2 mg/kg/d（根据病情调整剂量）。同时应避免使用可能加重肌无力的药物（如β-受体阻滞剂、含镁离子药物、喹诺酮类、氨基糖苷类及大环内酯类抗生素等）。治疗效果不佳时免疫球蛋白0.4 g/kg/d或者血置换，连续5 d。治疗过程中需要密切注意肺功能、神经系统症状变化；③无菌性脑膜炎：3级以上需要永久停止ICIs，住院治疗。在脑脊液结果明确以前，考虑静脉注射无环鸟苷直至获得聚合酶链反应（polymerase chain reaction, PCR）结果，或静脉给予阿昔洛韦抗病毒治疗。除外细菌和病毒感染，密切监控而不使用GC。如果出现中重度症状，试验性应用甲基泼尼松龙1 mg/kg/d-2 mg/kg/d治疗脑炎。如诊断明确甲基泼尼松龙1 mg/kg/d-2 mg/kg/d，如果症状严重或者出现寡克隆带，给予甲基泼尼松龙，1 g/d，连续3 d-5 d，即GC增量。同时给予免疫球蛋白，0.4 g/kg/d，连续5 d；④横断性脊髓炎：永久停止ICIs，请神经内科会诊，给予甲基泼尼松龙2 mg/kg/d，根据病情，可给予冲击剂量甲基泼尼松龙1 g/d，连续3 d-5 d，免疫球蛋白0.4 g/kg/d，连续5 d，或者血浆置换^[34]^。

### 4.4 免疫相关性皮肤不良反应

根据现有指南/共识，危重型皮疹定义为大疱性皮炎、Stevens-Johnson综合征（Stevens Johnson syndrome, SJS）即中毒性表皮坏死松解症（SJS/toxic epidermal necrolysis, SJS/TEN）和伴嗜酸粒细胞增多和系统症状的药疹（drug rash with eosinophilia and systemic symptoms, DRESS），多为3级-4级毒性，并可同时出现。治疗策略包括永久停用ICIs，泼尼松/甲基泼尼松龙1 mg/kg/d-2 mg/kg/d，需要住院治疗。

在真实世界中，银屑病、血管炎伴紫癜性皮疹等均可呈危重和难治性，因此鼓励患者一出现症状即进行报告。治疗除考虑泼尼松外，硫唑嘌呤、霉酚酸酯、甲氨蝶呤、四环素类抗生素（四环素、多西环素、米诺环素等）、达普松和烟酰胺可作为有效的类固醇替代药物。对于急性期严重银屑病，可考虑应用抗TNF-α、IL-1阻断剂、抗IL-23和抗IL-12单抗。皮肤血管炎推荐应用抗CD20单抗^[34]^。

### 4.5 免疫相关性消化系统不良反应

危重和难治性消化系统毒性包括肝脏毒性、胰腺毒性（急性胰腺炎）、胃肠毒性（腹泻/结肠炎），此外，也有关于食道炎、胃炎、十二指肠炎和小肠炎、胆管炎及胰管炎的个案报道。

肝脏毒性：通常表现为无症状性免疫相关肝炎，表现为谷丙转氨酶或谷草转氨酶升高，合并或不合并胆红素升高，大部分患者伴有发热。总体的治疗原则是，静脉使用GC、霉酚酸酯和他克莫司或专科医师指导下治疗。若肝酶及症状好转，可考虑GC逐步减量；若观察2 d-3 d，疗效不佳时，ATG作为GC无效时的推荐治疗，不推荐英夫利昔单抗（大多以风险过大提出警戒）。建议永久停用免疫治疗。在同一类ICIs中不提倡换用，在不同类ICI中可以慎重考虑。此外，已有应用IL-6单抗、抗CD20、抗TNF-α单抗成功的案例，最佳治疗尚有待进一步探索。

胰腺毒性（急性胰腺炎）：治疗建议GC及专科治疗，疗效差可考虑霉酚酸酯。由于目前报道的病例数少，治疗经验不足，GC和霉酚酸酯以外的治疗尚无明确推荐，但根据irAE的发病机理，特异性免疫抑制治疗值得进一步探索。

胃肠毒性：胃肠毒性主要表现腹泻/结肠炎，一般发生在平均3次治疗之后，也可能发生在紧随第一次治疗之后或免疫治疗中止后的数月，临床表现类似于慢性炎症性肠病。接受免疫联合治疗的患者中，3度-4度腹泻发生的风险要高于免疫单药治疗。建议对于初发初治、临床典型的3度-4度腹泻，无需等待肠镜检查即可开始GC。如48 h GC无效，可考虑加用英夫利昔单抗；如果仍无效，可考虑α4β7整合素拮抗剂维多珠单抗治疗，少有建议霉酚酸酯。欧洲肿瘤内科学会指南（European Society for Medical Oncology, ESMO）指南不排斥止泻药应用，其他指南未做建议。对于长期使用GC效差的难治性腹泻，建议需要尽快进行肠镜检查，首先排除感染，抗TNF-α单抗效果不佳时还可以尝试使用IL-6R抑制剂、IL-1抑制剂、IL-17单抗、IL-23单抗及IL-12单抗。必要时需外科手术引流术和穿孔修复术^[34]^。

**Table:** 《肺癌围手术期免疫治疗相关不良反应管理的临床诊疗建议》编审委员会成员名单（按姓氏汉语拼音排名）

	单位	姓名
组长	中国医学科学院北京协和医院	张力
组长	北京大学肿瘤医院	吴楠
特邀顾问专家	复旦大学呼吸病研究所	白春学
特邀顾问专家	中国人民解放军总医院	陈良安
特邀顾问专家	北京大学国际医院	梁军
特邀顾问专家	中国肺癌杂志	刘谦
特邀顾问专家	广东省人民医院	吴一龙
特邀顾问专家	中国医学科学院肿瘤医院	王洁
特邀顾问专家	中国医学科学院北京协和医院	张奉春
特邀顾问专家	中国医学科学院北京协和医院	张抒扬
顾问专家	福建医科大学附属协和医院	陈椿
顾问专家	天津医科大学总医院	陈军
顾问专家	上海市胸科医院	方文涛
顾问专家	中国医学科学院肿瘤医院	高树庚
顾问专家	浙江大学医学院附属第一医院	胡坚
顾问专家	空军军医大学第二附属医院	姜涛
顾问专家	中国医学科学院北京协和医院	李单青
顾问专家	上海交通大学医学院附属瑞金医院	李鹤成
顾问专家	华中科技大学同济医学院附属协和医院	廖永德
顾问专家	中国人民解放军总医院	刘阳
顾问专家	中日友好医院	刘德若
顾问专家	中国医科大学肿瘤医院辽宁省肿瘤医院	刘宏旭
顾问专家	吉林省肿瘤医院	刘建阳
顾问专家	四川大学华西医院	刘伦旭
顾问专家	中国医学科学院北京协和医院	王孟昭
顾问专家	天津医科大学附属肿瘤医院	王长利
顾问专家	北京大学人民医院	杨帆
顾问专家	北京大学肿瘤医院	杨跃
顾问专家	中山大学附属肿瘤医院	张兰军
顾问专家	首都医科大学宣武医院	支修益
顾问专家	广东省人民医院	钟文昭
执笔人	中国医学科学院北京协和医院	倪军
执笔人	北京大学肿瘤医院	黄淼
执笔专家	中国医学科学院北京协和医院	管宇宙
执笔专家	中国医学科学院北京协和医院	郭潇潇
执笔专家	中国医学科学院北京协和医院	何春霞
执笔专家	中国医学科学院北京协和医院	李玥
执笔专家	北京大学肿瘤医院	李少雷
执笔专家	中国医学科学院北京协和医院	梁乃新
执笔专家	北京大学肿瘤医院	鲁方亮
执笔专家	北京大学肿瘤医院	吕超
执笔专家	中国医学科学院北京协和医院	吕玮
执笔专家	中国医学科学院北京协和医院	斯晓燕
执笔专家	中国医学科学院肿瘤医院	谭锋维
执笔专家	中国医学科学院北京协和医院	王汉萍
执笔专家	中国医学科学院北京协和医院	王江山
执笔专家	北京大学肿瘤医院	阎石
执笔专家	中国医学科学院北京协和医院	杨华夏
执笔专家	中国医学科学院北京协和医院	朱惠娟
执笔专家	中国医学科学院北京协和医院	庄俊玲
执笔专家	北京大学肿瘤医院	卓明磊
秘书	中国医学科学院北京协和医院	崔晓霞
